# Investigation of the Ultrasonic Treatment-Assisted Soaking Process of Different Red Kidney Beans and Compositional Analysis of the Soaking Water by NIR Spectroscopy

**DOI:** 10.3390/s25020313

**Published:** 2025-01-07

**Authors:** Matyas Lukacs, Tamás Somogyi, Barasa Mercy Mukite, Flóra Vitális, Zoltan Kovacs, Ágnes Rédey, Tamás Stefaniga, Tamás Zsom, Gabriella Kiskó, Viktória Zsom-Muha

**Affiliations:** 1 Department of Food Measurement and Process Control, Institute of Food Science and Technology, Hungarian University of Agriculture and Life Sciences (MATE), Somlói út 14-16., H-1118 Budapest, Hungary; lukacs.matyas.krisztian@phd.uni-mate.hu (M.L.); somogyi.tamas.3@phd.uni-mate.hu (T.S.); mukite.barasa.mercy@phd.uni-mate.hu (B.M.M.); vitalis.flora@uni-mate.hu (F.V.); kovacs.zoltan.food@uni-mate.hu (Z.K.); redey.agnes@phd.uni-mate.hu (Á.R.); stefaniga.tamas@phd.uni-mate.hu (T.S.); zsomne.muha.viktoria@uni-mate.hu (V.Z.-M.); 2 Department of Postharvest, Supply Chain, Commerce and Sensory Science, Institute of Food Science and Technology, Hungarian University of Agriculture and Life Sciences (MATE), Ménesi út 43-45., H-1118 Budapest, Hungary; zsom.tamas@uni-mate.hu; 3 Department of Food Microbiology, Hygiene and Safety, Institute of Food Science and Technology, Hungarian University of Agriculture and Life Sciences, Somlói út 14-16., H-1118 Budapest, Hungary

**Keywords:** NIR spectroscopy, chemometrics, canning, pulse, technology, hydration

## Abstract

The processing of beans begins with a particularly time-consuming procedure, the hydration of the seeds. Ultrasonic treatment (US) represents a potential environmentally friendly method for process acceleration, while near-infrared spectroscopy (NIR) is a proposedly suitable non-invasive monitoring tool to assess compositional changes. Our aim was to examine the hydration process of red kidney beans of varying sizes and origins. Despite the varying surface areas, the beans’ soaking times of 13–15, 15–17, and 17–19 mm did not reveal significant differences between any of the groups (control; low power: 180 W, 20 kHz; high power: 300 W, 40 kHz). US treatment was observed to result in the release of greater quantities of water-soluble components from the beans. This was evidenced by the darkening of the soaking water’s color, the increase in the a* color parameter, and the rise in the dry matter value. NIRs, in combination with chemometric tools, are an effective tool for predicting the characteristics of bean-soaking water. The PLSR- and SVR-based modelling for dry matter content and light color parameters demonstrated robust model fits with cross and test set-validated R^2^ values (>0.95), suggesting that these techniques can effectively capture the chemical information of the samples.

## 1. Introduction

The initial stage of the processing dry pulses, including red kidney beans, is the soaking. While soaking, water diffuses into the legume and moves towards its center. Thus, such a water absorption process makes the legume develop a uniform texture [1]. The water that enters the crop plays a role in facilitating the process of starch gelatinization and protein denaturation during the blanching process [2]. Soaking removes nutritional factors (e.g., lectins, phytic acid) from the crop and reduces cooking time [2]. However, during the soaking process, a considerable quantity of water-soluble antioxidants is dissolved from the crop [2]. Fernandes et al. (2010) [3] suggest that soaking prior to cooking yields more favorable outcomes than unfavorable ones. Soaking the beans is not only nutritionally important but also an important part of the production process, as it reduces cooking time and affects the quality of the final product. However, this process is a time-consuming procedure, which affects the speed of the production process. Therefore, many studies have been done to speed up water absorption. Increasing the soaking temperature is one of the most obvious techniques to reduce the viscosity of water, thus helping the diffusivity of water by enlarging the size of the bean pores, thereby increasing the speed of water absorption [4].

One disadvantage of soaking at high temperatures is that it also promotes thermal degradation of bioactive compounds and reduces their moisture balance [4]. In addition to the degradation of bioactive compounds, a high amount of energy is required to provide higher temperatures, which also results in higher production costs [5]. Naviglio et al. (2013) [6] used cyclic pressure during soaking to hydrate cannellini beans, which increased the penetration of liquid inside the cells and, thus, significantly reduced hydration time. A reduction in hydration time has been observed during high-pressure soaking [7], or even during ultrasound-assisted hydration processes [8,9,10].

Ultrasound (US) is a mechanical sound wave above the audible frequency range, i.e., above 20,000 Hz [11]. Within ultrasound, we distinguish between low-power (high frequency) and high-power (low frequency) ultrasound. Low-power ultrasound can be used to determine the quality parameters of food, while high-power ultrasound treatment causes changes in the treated sample due to cavitation. Such changes can be physical, chemical, or biochemical [12]. Ultrasound treatment results in cavitation. Bubbles are formed in the fluid, which are subjected to lower pressure than the surroundings during the rhythmic part of the cycle, which then expand and collapse due to the high stress in the walls [13]. The collapse results in high pressure and temperature in the tiny space around the bubble, creating an extreme physical environment under the right conditions, making it suitable for food-technological applications [14].

In a watery medium (like soaking water), the ultrasound-generated cavitation facilitates the initiation of pores and surface erosion of the skin, allowing for the influx of water into the tissue and resulting in the swelling phenomenon. On the other hand, parallel to swelling, the extraction of internal compounds via leaching is also accelerated due to changes in the structure of the semipermeable membrane system of the peel tissue structure [15,16]. Cavitation induces physical and chemical/biochemical alterations in the sample, thereby facilitating a number of food processing operations [12], such as extraction [17], filtration, foaming, freezing, crystallization, cutting, and reduction of the bacterial count (e.g., sterilization, pasteurization) [12,18,19].

The use of ultrasonic treatment has a significant impact on the speed of various processes in the food industry. Ultrasound can be used to reduce processing costs, using a shorter time and lower energy consumption than is normally required for conventional processes. Ultrasound-induced food processes are influenced partly by cavitation phenomena and enhanced mass transfer [19,20]. Miano et al. (2018) [9] showed that higher temperatures and ultrasound separately improve the hydration process in the beans. In contrast, warm temperatures inhibit the effect of ultrasound. Ulloa et al. (2015) [10] also investigated the effects of ultrasound and found a reduction in soaking time and cooking time with the addition that the extent of the reduction also depends on the bean type and the exposure time of ultrasound.

Similarly to recent food-technological developments, analytical measurement techniques ensuring food quality at various steps of production have also expanded noticeably. Near-infrared spectroscopy (NIRS) plays an increasingly important role in quality and quantity control in the food industry [21]. NIRS is a non-destructive analytical method that, like Raman spectroscopy, is based on the extraction of information from the vibrational excitation of molecules [22]. NIR spectroscopy, as a non-invasive analytical technique, offers several advantageous features. In comparison to conventional chemical methods, it does not necessitate the time-consuming sample preparation, thus reducing the overall time and effort required while minimizing environmental impact. Moreover, it can be integrated effectively into food processes [23], which can play an integral role in the quality control process from production to the finished product.

In a recent study, Boadu et al. (2024) [24] demonstrated the efficacy of near-infrared (NIR) spectroscopy in differentiating various African coffee varieties. They have also developed a system that can assist in the detection of food fraud in coffee beans. Jiang et al. (2025) [25] demonstrated that NIR spectroscopy can be employed for the accurate prediction of the fermentation state of 3D-printed dough. In a study conducted by Qiu et al. (2022) [26], NIR spectra were employed in combination with multivariate calibration models to predict the antioxidant capacity of edible rose flowers during infrared drying.

NIR technology will play an important role in the future of digitalized and sustainable food production [27]. NIR spectroscopy allows continuous monitoring during the production process resulting in the development of precise process controls by the industry. As an overall result, individual processes will be optimized and better-quality food will be produced. Therefore, more and more rely on upcoming technological developments of NIR equipment aiming to maximize the industrial application and efficiency increase [28,29].

NIR spectrometry is a technique based on the absorption of electromagnetic radiation, which measures in the near-infrared range (approximately 700–2500 nm) [30]. In the measurement, a variety of food raw materials, semi-finished products, and finished products are irradiated at different near-infrared wavelengths, and the reflected or transmitted radiation is subsequently measured. Particular organic molecule absorption in the NIR region can explain the chemical composition of the sample being analyzed if correlated to data from primary analytical methods using chemometric tools [31]. From these processes, it is possible to predict the chemical components present in the sample under investigation [32], as well as additional microstructural properties such as stiffness and internal damage [33].

The use of NIR spectroscopy to gain a deeper understanding of complex biological systems is becoming increasingly prevalent [32]. Recent research has demonstrated the suitability of NIRS for real-time monitoring of diverse food processes [34]. However, the majority of studies have been conducted in laboratory settings, with only a limited number of potential applications at an industrial level [29]. Wafula et al. (2021), in their studies [35,36], employed NIRS technology to predict the cooking time of beans and predict the onset of hard-to-cook defects.

Furthermore, NIRS can be employed to examine the molar ratios of minerals and antinutrients in raw ground beans [37]. In the case of chickpea drinks, NIR spectroscopy could be employed to provide a quantitative prediction of phytochemicals [38]. NIRS is a green technology with unique properties that will most likely play an important role in the future of digitalized and sustainable food production [27]. However, the complexity of NIR spectra can present a significant challenge in the interpretation of spectral data [39], which requires a thorough understanding and proper implementation of available chemometric tools.

Consequently, NIRS offers the potential to continuously monitor the leaching of useful components from beans into the liquid medium during the soaking process. This enables the automation of the measurement of quantitative and qualitative parameters of selected components. However, there is no accessible study that uses NIRS to determine the composition of the soaking water, which could provide an indirect but valuable insight into the compositional changes of the beans, assuming an interdependent system.

The application of ultrasound treatment has been demonstrated to facilitate the process of bean hydration. However, it also accelerates the dissolution of water-soluble substances present in the bean in the soaking water. As the substances that dissolve from the beans are transferred to the soaking water, it is possible to analyze the soaking process by analyzing the composition of the soaking water. In this analysis, NIRS represents a novel method that provides non-invasive monitoring of the process.

Therefore, the objectives of this study were

- To evaluate the impact of ultrasound-assisted soaking on the soaking time and leaching behavior of red kidney beans of varying sizes and origin;
- To assess the feasibility of using near-infrared spectroscopy (NIRS) to monitor the leaching process during ultrasonic treatment;
- To investigate the impact of ultrasound-assisted soaking on the leaching behavior of red kidney beans. In particular, the study will examine how ultrasonic soaking affects the amount of leached material and determine whether accelerating the lengthy soaking process in industrial processing with ultrasound will result in a significant change in the leaching process of beans.

## 2. Materials and Methods

### 2.1. Materials

The soaking experiments were conducted on red kidney beans (*Phaseolus vulgaris* cv. *Rampart*) of varying geographical origins, including the United States of America (USA) and the Slovak Republic (Slovakia). The beans of American origin were sourced from the Ciacam Sa grocery store (Vitrolles, France), while the Slovakian beans were obtained from GAMOTA VD (Komárno, Slovakia). The beans, originating from different geographical locations, were transported and stored under the same environmental conditions, thereby minimizing the potential impact of external factors on the quality of the raw material.

The beans in bulk exhibited variation in length, with measurements ranging from 11 mm to 19 mm. Subsequently, the beans were sorted according to their length. The beans were manually sorted into four size categories, designated as follows: S (small, <13 mm); M (medium, 13–15 mm); L (large, 15–17 mm); and X (extra-large, >17 mm). The minimum number of beans required for the smallest size category (S) was not available in the sample obtained, which made it impossible to perform parallel measurements.

### 2.2. Ultrasonic Treatment

An ultrasonic bath with a capacity of 16 L (HBM Machines, Moordrecht, The Netherlands) was employed to assist the soaking of the beans. Considering the characteristics of the US device used, the upper and lower limits of the frequency and power settings were chosen for the experiments (20 and 40 kHz; 180 and 300 W). Two distinct ultrasound treatments were applied to the treated groups according to the features of the device: a low-power (LP, 20 kHz, and 180 W) and high-power (HP, 40 kHz, and 300 W) treatment.

From each group, 20 g of beans were weighed into 200-mL glass beakers, and 120 g of tap water were added to the beakers. To ensure optimal conductivity of the ultrasound, the apparatus was filled two-thirds up with tap water, and the beakers were placed within this medium. To avoid any thermal effects during the ultrasonic treatment, an external thermostat was connected to the system in order to ensure a constant flow and a constant water temperature of 25 ± 1 °C throughout the full duration of the treatment. The control samples were exposed to the same temperature conditions as the samples that had been treated with ultrasound. The same experimental setup was used for all size classes.

### 2.3. The Hydration Process

Two separate experiments were conducted. In the first experiment, beans of different sizes and origins were soaked for 4 h at 25 °C for both the two levels of ultrasonic treatment and the control.

In accordance with industrial practice, the increase in weight of the crop during soaking was used to determine the soaking time of the beans for both the control and treated groups. The soaking process was considered complete when the initial weight of the beans had doubled. This is the stage in the industry where the beans, after the soaking process, are ready to go through further manufacturing process.

During the experiment, the weight of the beans was recorded every 30 min.

In the second experiment, hydration was stopped for the different groups when the sample had reached twice its initial weight.

Combining the two experiments, a total of 66 groups were tested.

The change during the process of hydration was modeled using the exponential equation. The general form of this equation is provided in Equation (1).

(1) $$Swelling\;rate\;value\;(SRV)=\left(1-{SRV}_{final}\right)\cdot e^{-\frac{t}{\tau }}+{SRV}_{final}$$

The *SRV* final value represents the endpoint of the soaking curve, *t* is the soaking time, and tau (*τ*) is the time constant that characterizes the soaking dynamics. The model parameters permit a numerical comparison of the hydration processes of the various groups.

### 2.4. Soaking Water Sample Preparation

Roughly 100 mL of soaking water/sample were available following the ultrasonic treatment of beans. Samples were derived from beans belonging to the four size groups, with three replicates for each sample, except the smallest size. In the case of the smallest size group, the bulk sample did not contain sufficient volume to measure in three replicates. So, in that case, only two measurements were performed for each treatment group. For the control group, three replicates were used. To minimize differences in chemical degradation across samples, each sample was immediately frozen after the soaking process.

In order to compare the ultrasonically treated and control samples, the soaking water of the bean samples in Experiment 2 (soaked to twice the mass) was also tested. In all cases, this resulted in a reduced soaking time for the ultrasonically treated samples compared to the control. Apart from the difference in treatment times, the new sample set was prepared and measured in the same way as the original sample set in the first experiment.

### 2.5. Near-Infrared Spectroscopic Measurements

For the analysis of bean soaking water, the samples were subjected to near-infrared scanning, and the sample spectra were collected using NIRS XDS instrument equipped with a Rapid Liquid Analyzer (RLA) module (Metrohm, Herisau, Switzerland) with 1 mm path length open top quartz cuvette in the 400–2500 nm spectral range. Spectral acquisition of all the samples was performed at constant room temperature. This, together with relative humidity, was monitored using Voltcraft DL-121TH multi-data logger (Conrad Electronic, Berlin, Germany). The cuvette was tempered at 30 °C, and samples were incubated in the cuvette for 90 s before measurement. Prior to measurements, the frozen samples were defrosted in a 45 °C water bath for 16 min. The order of sample analysis was randomized. Three consecutive spectra were recorded per sample, and a reference/background scan (Milli-Q water) was measured after every fifth sample.

### 2.6. Color Measurement

Colorimetric measurements of the soaking water of treated beans were done in CIE-L*a*b* tristimulus color coordinate system by ColorLite sph850 spectrophotometer (ColorLite GmbH, Katlenburg-Lindau, Germany) using transmission mode. The instrument records the spectra from 400–700 nm in 10 nm steps with the standard probe head. The bean-soaking water was filled into the cuvettes, and the measurement was conducted using the CA10-LS cuvette holder attachment. Before the measurement, the device was calibrated using distilled water.

The Euclidean distance between the two sample colors in CIE-L*a*b* space can be calculated using the following equation, Equation (2) [40]:

(2) $$∆{E^{*}}_{ab}=\sqrt{(∆L^{*}{)}^{2}+(∆a^{*}{)}^{2}+(∆b^{*}{)}^{2}}$$

where:

ΔE*_ab_ is the value of the total color difference;

ΔL*, Δa*, Δb* is the CIE-L*a*b* color difference value of the two-color data.

In the first step of the analysis, the color change of the treated samples was compared to the untreated (control) samples. In the next step, the samples treated at low ultrasonic level were compared to the samples treated at high level.

The color of the soaking water samples was measured in transmission mode. The final L*a*b* values were the average of three measurement repetitions per sample.

### 2.7. Dry Matter Measurement

Dry matter content (DMC, %) was determined for each of the soaking water samples as well, using the conventionally applied drying chamber method. A sample of about 18 g was weighed and dried for 12 h at 105 ± 1 °C to constant weight [41]. The wet and the dry weight of the samples were measured with an analytical scale. The DMC (%) was calculated according to Equation (3).

(3) $$DMC\left[\%\right]=\frac{W_{n_{12}}}{W_{n_{0}}}\times 100\;$$

where:

$W_{n_{0}}\;$ is the initial net weight of a certain sample solution;

$W_{n_{12}}\;$ is net weight of a certain sample solution after 12 h of drying.

### 2.8. Data Analysis

Results of reference measurements were processed with MS Excel (2016). Exponential model fitting was performed using the Solver add-in. SPSS for Windows ver. 14.0 statistical software was used to evaluate measurement data. ANOVA statistical analysis was performed at 95% significance level (α = 0.05). For post hoc analysis, Tukey test was used.

R-project (v. 4.3.0, 2023, The R Foundation for Statistical Computing, Vienna, Austria; using R package: aquap2 [42]) and MS Excel (Microsoft Corporation, Redmond, WA, USA) were applied for multivariate data evaluation. To reduce baseline shift and slope differences caused by unwanted variance and spectral noise, a combination of Savitzky–Golay (SG) smoothing filter, multiplicative scatter correction (MSC), standard normal variate (SNV), and detrending (deTr) were tested to pre-treat the spectra before further evaluation [43]. These methods have all shown positive impacts on modeling performance and robustness in multiple previous studies [44,45,46]. Optimal pre-treatment combinations were selected by observing patterns on raw spectra and PCA score plots, followed by additional tuning during supervised modeling by evaluating changes in model metrics and corresponding regression vectors [47].

To identify the interrelationships between the samples and to detect outliers, principal component analysis (PCA) was performed [48]. PCA was also used to provide input variables for support vector regression (SVR)-based modelling, as it was previously reported as a reliable variable selection tool to improve model robustness for techniques that do not inherently incorporate dimension reduction [49]. Partial least squares (PLSR) and support vector regression (SVR) were used to derive calibration models to predict dry matter content and colorimetric (L*, a*, b*) values after the detection and removal of outliers using PCA. PLSR is a widely used method that integrates elements of principal component analysis and multiple regression. It aims to predict or explore a set of dependent variables using a group of independent variables or predictors. This is accomplished by deriving a set of orthogonal components, known as latent variables, from the predictors, which maximize predictive performance [50].

Support vector regression (SVR), similarly to PLSR, identifies a linear relationship between the independent variables and dependent variables. Its cost function, which is minimized to achieve the optimal regression model, includes a two-norm penalty on the regression coefficients, an error term scaled by the error weight C, and a set of constraints. This cost function aims to minimize both the magnitude of the coefficients and the prediction errors, balancing smoothness and accuracy. Controlling the coefficient size is crucial, as excessively large coefficients can reduce generalization ability by increasing variance, leading to overoptimistic predictions [51]. The methods were selected due to their applicability for small sample sets and the assumption that soaking water samples are relatively simple matrices that would not produce serious non-linear patterns between spectral and reference data [52] and warrant the use of more sophisticated learning algorithms.

The use of additional kernel functions provided by SVR to account for potential non-linearities was deemed sufficient for the present sample set. These methods have proven applicable to predict phenolic compounds and macronutrients in a wide variety of biological matrices with comparable performance in the past [51,53,54].

SVR and PLSR models were cross-validated, leaving one replicate (three consecutive together) out. [55]. Test-set prediction was performed to test the robustness of the models by using 80% of the data for training and 20% for predicting, repeated three times with a different prediction set, and selected representatively.

Additionally, SVR models were optimized by selecting the number of input PCs and simultaneously tuning hyperparameters. This tuning process included adjusting the error penalty parameter (C: 0.1–10) and the maximum permissible error (ε: 0.01–0.5) while also evaluating various kernel functions (linear, radial, polynomial, and sigmoid) [49,51].

To prevent overfitting, a maximum allowable difference of ~15% was set between the calibration and validation average error values. The tendency and difference of error values for calibration and validation based on the number of components used were followed by applying scree-plots. For models using a linear kernel, similarly to the case of PLSR, regression vectors were additionally visualized to identify the presence of noise and the potential modeling of unwanted variance resulting in too specific, generally inapplicable models [56]. As a rule of thumb, the number of components used for PLSR and SVR modeling were also kept under 1/10th of the sample set to further minimize the risk of overfitting [55].

The average and standard deviation of model parameters, including the determination coefficient of calibration (R2c), root-mean-square error of calibration (RMSEC), determination coefficient of cross-validation (R2cv), root-mean-square error of cross-validation (RMSECV), determination coefficient of prediction (R2p), and root-mean-square error of prediction (RMSEP) were calculated and used to evaluate model performance.

## 3. Results

### 3.1. Analysis of the Soaking Process

**Figure 1:**
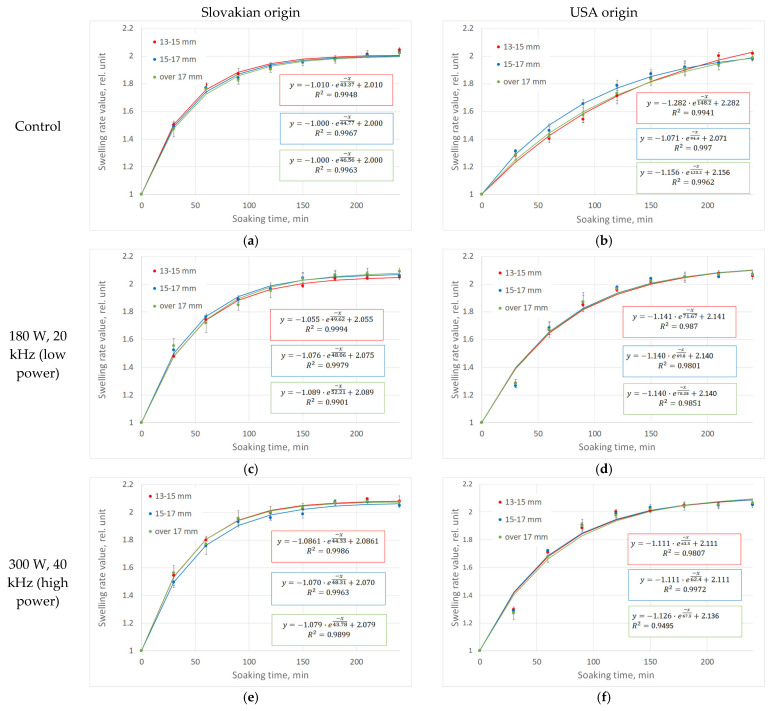
The change of swelling rate value of red kidney beans with Slovakian and USA origin (**a**,**b**) control; (**c**,**d**) 180 W, 20 kHz ultrasonic treated (LP) and (**e**,**f**) 300 W, 40 kHz (HP) ultrasonic treatment sample) versus soaking time in relation to average bean size (±SD) (marked with different colors).

In the first experiment, the different groups were soaked for a 4 h period for each treatment. The objective of this experiment was to analyze and monitor the soaking process. The hydration process of beans from different growing locations and sizes was initially examined. Figure 1 illustrates the change in beans’ weight relative to their original weight (SRV—swelling rate value) as a function of soaking time in minutes (min). It can be observed that the hydration process for all size groups and origins could be well approximated by an exponential equation.

The data obtained from the first experiment were employed in the analysis. Figure 1 illustrates the mean and standard deviation of the parallel mass measurements, as well as the exponential model that was fitted to the mean swelling rate values of the soaking process for different sizes and origins. The SRV 2 state is characterized by a doubling of the original mass of the bean due to soaking. At this stage, the bean is prepared for processing, which, in industrial practices, signifies the conclusion of the soaking process.

For all sizes, for both types, it was observed that ultrasound treatment, regardless of the power and frequency used, has a positive effect on the weight increase of the beans during soaking time. Furthermore, it can be seen in Figure 1 that the soaking tendency of the beans was different for the two origins. In the case of the Slovakian bean, the weight increase was more rapid at the beginning of the process, while in the case of the USA bean, a much slower process was observed.

The results obtained can be employed to calculate the requisite time in min to reach SRV 2, which represents the soaking time needed to process the beans. This may be achieved by fitting a curve to the soaking process. The soaking times were then compared for the various size groups that were tested, and the results are presented in Table 1.

The ANOVA analysis of the results indicates that there was no statistically significant difference (*p* < 0.05) in the soaking time required for processing for any of the size classes or treatments or origins. Therefore, based on the presented data, it can be concluded that, despite the different sizes and, thus, surface area and weight of the beans tested, there is no significant difference in the technological process for either conventional or ultrasound-assisted soaking for the size categories tested (13–17 mm).

This observation has the potential to be a significant outcome for industrial practice, as it eliminates the necessity for size presorting prior to soaking. In the size range under investigation, the hydration of beans soaked by both ultrasonic and conventional methods would be uniform. This is an important factor for further heat treatment and has an impact on the quality of the canned product.

### 3.2. Analysis of the Effects of Treatment

**Figure 2:**
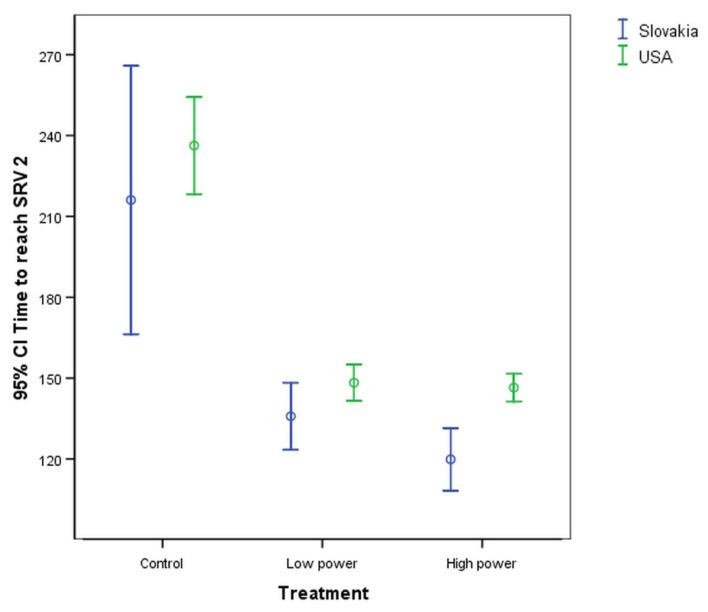
Time required to reach SRV 2 for different treatments for samples from the two different growing areas.

The results obtained from examining the different treatment groups are presented in Figure 2, which shows the average time and its 95% confidence intervals required to reach SRV 2. Figure 2 demonstrates that both types of US-assisted soaking treatment significantly reduce the soaking time (*p* < 0.05) compared to the control. This finding is consistent with the already published results reported by [10,57].

Additionally, based on the results of the Tukey test, no significant difference was observed between the values obtained for the low (180 W, 20 kHz) and high (300 W, 40 kHz) treatment levels for either of the samples from the growing area. The results indicated that the higher-powered treatment did not result in a significant reduction in the required treatment time when compared to the applied lower-powered treatment.

### 3.3. Examining the Effect of Growing Area

It is valuable to have data regarding the soaking process of beans from different geographical regions, as it is important to understand the influence of technology on the processing procedure. Figure 1 illustrates that the dynamics of the soaking process differ between beans from the two growing areas. This can be examined numerically by studying the parameters of the fitted exponential curve.

**Figure 3:**
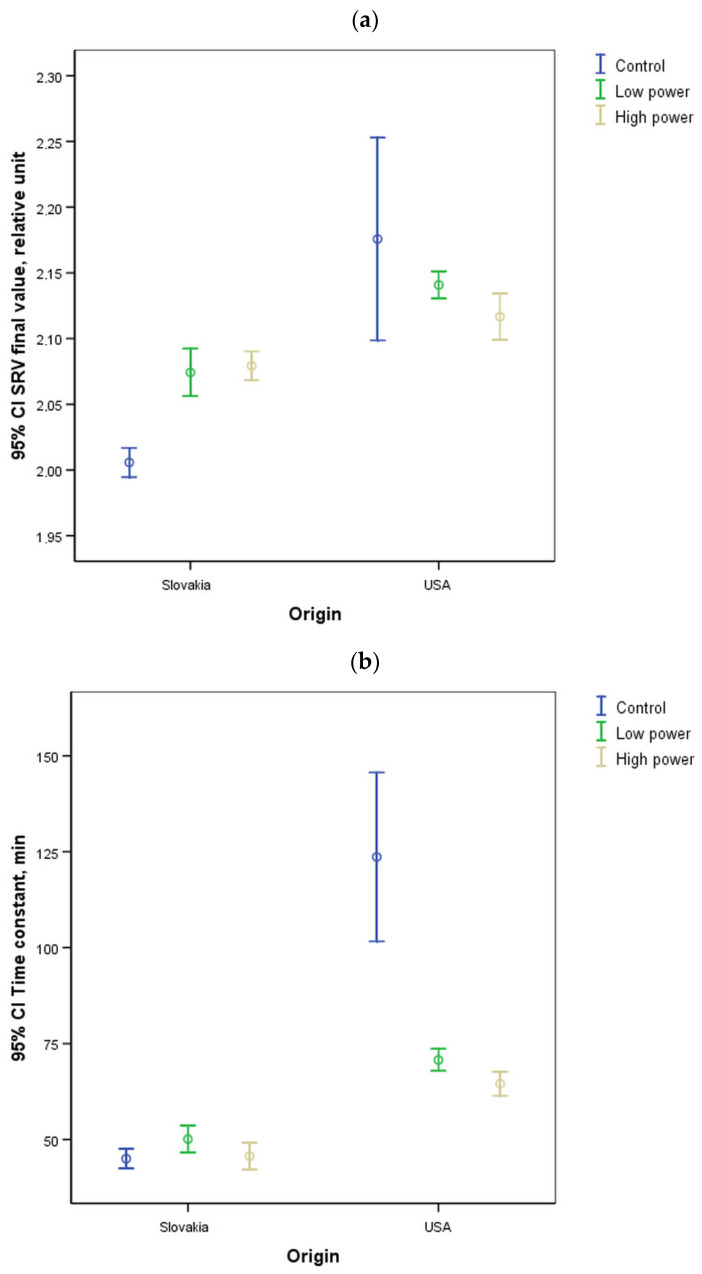
Comparison of the parameters of the soaking process curve for bean samples from different growing areas (**a**) final SRV value, (**b**) a tau [τ] value representing the speed of the process.

Figure 3 illustrates the mean and 95% confidence intervals of the time constant (τ) and SRV final values of the samples from the disparate growing regions. From Figure 3, it can be seen that the SRV final and tau (τ) of two samples from two growing areas are significantly different for both untreated and two types of US treatments. The difference in tau values (τ) indicates disparate hydration dynamics. In Slovakian beans, the hydration process was more rapid at the outset than in beans of USA origin.

Having compared the soaking time needed to reach the canning industry-desired SRV 2 level in the case of normal and ultrasound-aided soaking processes, by the use of ultrasonic treatments with different levels of intensity, a significant soaking time decrease can be reached. Based on the data shown in Table 1, it can be concluded that the ultrasound treatment (low or high level) significantly accelerated the soaking process for bean samples in the case of both origins. Instead of the 3.5 (Slovakian) and 4 (USA) hours of soaking measured for the control sample, it took an average of 2–2.5 h by applying ultrasonic treatment. The soaking time needed to double the initial bean mass value can be reduced by approximately 30–40%.

### 3.4. Results of NIR Spectroscopic Measurement

#### 3.4.1. Exploratory NIR Data Evaluation Based on PCA Results

**Figure 4:**
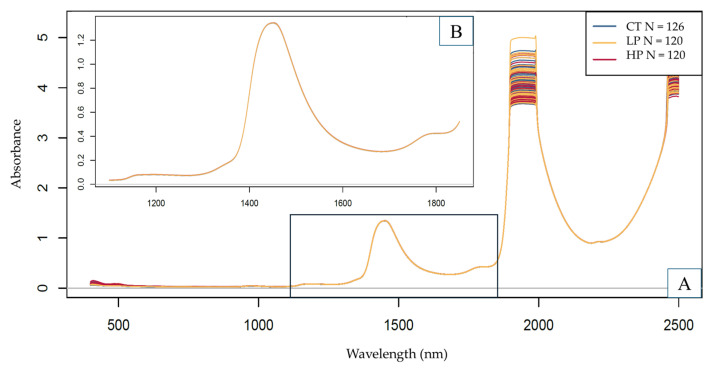
The NIR spectra are colored by the different treatment levels using the entire wavelength range (CT = control, LP = high-power, HP = high-power). The following markings were applied to the figure: (**A**) the entire wavelength range, (**B**) the selected wavelength range.

The NIR spectra colored by the different treatment levels using the entire wavelength range can be seen in Figure 4. By observing the raw spectra, the prominent region was identified to be at the intersection of the first and second overtones, around the 1400–1550 nm wavelength mark. This area is characterized by a wide range of molecular vibrations, including (aromatic) O-H, CH, and N-H bands, which could correspond to the presence of hydrocarbons, phenols, and protein derivatives [58].

Due to a detector shift at 1100 nm for the XDS device and, therefore, a visible baseline deviation, the range below 1100 nm was excluded. The region above 1850 nm, due to the strong presence of water, resulted in absorbance values too high to remain within the linear response range of the detector. Hence, this region was also omitted [59]. For these reasons, the 1100–1850 nm wavelength range was selected for further modeling. Since there was no sizeable baseline shift or slope difference in the reduced wavelength range of the recorded spectra (Figure 4B), the use of pre-treatment methods was generally kept at a minimum and individually adjusted in the case of each predictive model built.

**Figure 5:**
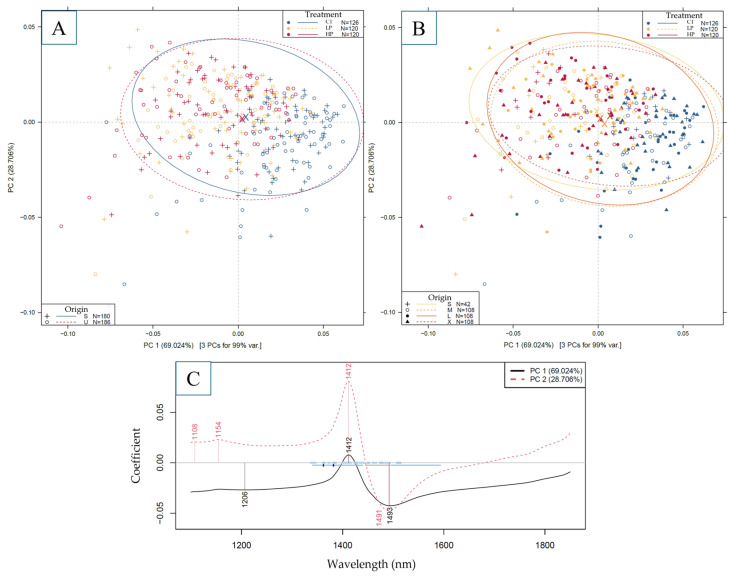
PCA score plots focusing on the separation of beans with different origins (**A**) and different size groups (**B**), with corresponding loading vectors (**C**). Marker colors are based on the treatment levels, while marker types and ellipsis colors are based on the origin (**A**) and size class (**B**) of the bean samples.

PCA results were summarized in Figure 5. By observing the score plots (Figure 5A,B), a separation of measurement points between the treated and the control group can be observed alongside the first principal component. By observing the loadings vector (Figure 5C), the prominent region responsible for this separation is between ~1400 and 1500 nm, in accordance with the observations on raw spectra (Figure 4). However, this separation is not clear between the two levels of ultrasonic treatment, nor between the different bean origins (Figure 5A) and size groups (Figure 5B). These observations imply a potential (chemical) difference between samples belonging to the different treatment levels, but not necessarily to samples belonging to different origins and size groups. Based on this, the entirety of the dataset without subdivision was used to build predictive models.

#### 3.4.2. Building Predictive Models Using PLSR and SVR

The cross and test set-validated PLSR and SVR models built for the prediction of dry matter and colorimetric values using the entire dataset are summarized in Table 2.

Dry matter content and lightness color parameter (L*) values were generally predicted accurately using both algorithms without the necessity to include multiple spectral pre-treatment methods, as deducted by observing the raw spectra. The highest R^2^P value in the case of DMC was 0.951 ± 0.008 with a corresponding RMSEP of 0.01% using SVR, slightly surpassing the results of PLSR. Similarly, in the case of L*, a slightly more favorable result was achieved using SVR compared to PLSR, reaching an R^2^P of 0.87 ± 0.012 and an RMSEP of 2.546 ± 0.122.

**Figure 6:**
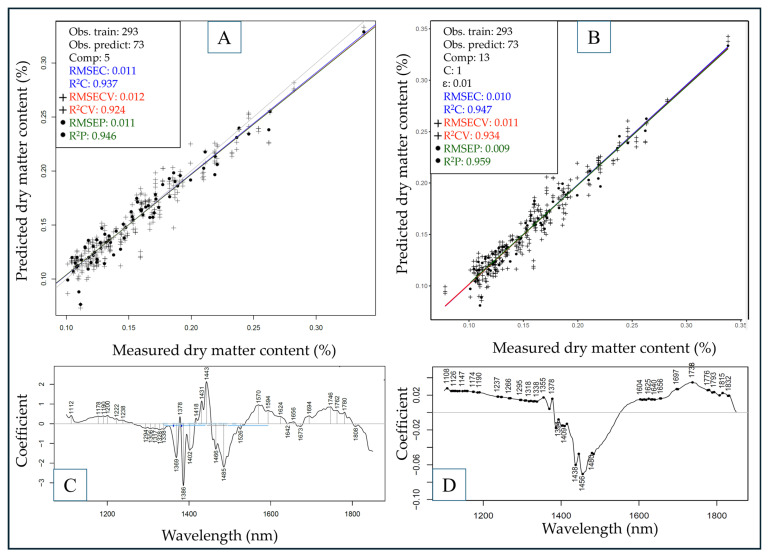
Models to predict DMC content built with PLSR (**A**) and SVR (**B**) with corresponding regression vectors (**C**,**D**). Savitzky–Golay filter with second-order polynomial and 21 smoothing points as pre-treatment for all models. Line colors and point marker types correspond to either calibration, cross-validation, or test-set validation (prediction), as indicated in the legend.

**Figure 7:**
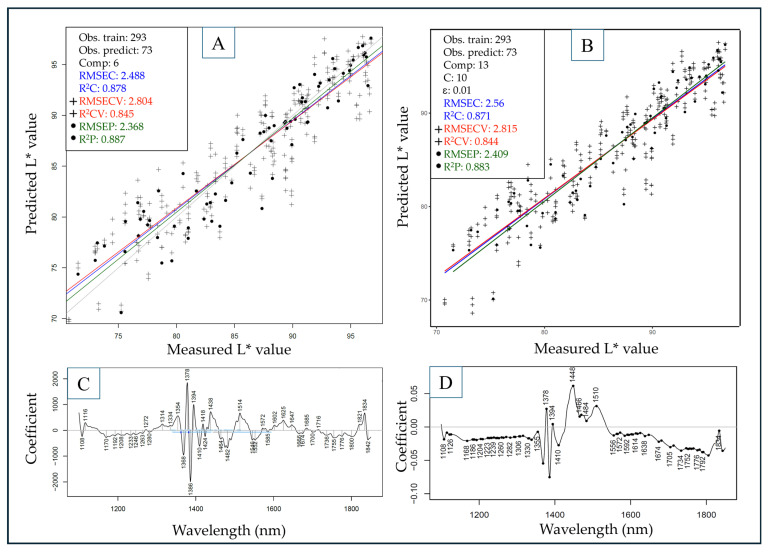
Models to predict L* values built with PLSR (**A**) and SVR (**B**) with corresponding regression vectors (**C**,**D**). Savitzky–Golay filter with second-order polynomial and 21 smoothing points as pre-treatment for all models. Line colors and point marker types correspond to either calibration, cross-validation, or test-set validation (prediction), as indicated in the legend.

For the prediction of a* and b* values, there was no unambiguous correlation between spectral and reference data, with the highest respective R^2^P and RMSEP values of 0.588 ± 0.134; 2.221 ± 0.349 in the case of a* and 0.573 ± 0.015; 4.507 ± 0.071 for b*. The much higher standard deviation between model metrics for a* and b* also implies worse generalization of these models, making them unreliable for predictions using different sample sets. However, it can be noted that in the case of a*, SVR proved to be much more feasible due to the flexibility of using different kernels (in this case radial) to account for non-linear patterns in the dataset. The best-performing models for DMC and L*, using both algorithms, are summarized in Figure 6 and Figure 7.

The best models to predict DMC content (Figure 6) could reach a respective R^2^P and RMSEP value of 0.946; 0.011% with PLSR and 0.959; 0.009% with SVR, proposing a similarly good performance for both algorithms. By observing the regression vectors (Figure 6C,D), the prominent wavelength region for the model predictions could be identified at ~1370–1500 nm with multiple notable peaks. The 1370–1400 nm area almost exclusively belongs to aromatic hydrocarbons and C–H stretchings in various aliphatic compounds. The numerous prominent peaks in the 1400–1500 nm region, more specifically at 1443 and 1485 nm, can primarily be attributed to O–H stretching and bending vibrations, particularly sensitive to moisture in samples. Apart from that, these peaks can additionally signal the presence of compounds with O–H, C–H, and N–H groups, like alcohols, phenols, proteins, and carbohydrates such as monosaccharides [58].

For the prediction of the lightness color parameter values (Figure 7), both algorithms performed similarly, with PLSR providing a model with slightly better metrics for the test set with an R^2^P value of 0.887 and an RMSEP of 2.368. Similar to the observations on raw spectra, loadings vectors, and regression vectors for DMC prediction, the prominent wavelength region contributing to model predictions could be identified at ~1370–1550 nm (Figure 7C,D). The 1500–1550 nm region, seemingly having more relevance than in the case of DMC predictions, stands almost exclusively for primary and secondary amines with N–H stretching and bending vibrations. This proposes a correlation between nitrogenous compounds and the lightness color parameter values of these samples.

As a summary, it can be concluded that both algorithms were found to be similarly feasible to build accurate predictive models for DMC and L* values, potentially capturing the chemical variance in the dataset without excessive model overfitting.

### 3.5. Testing the Effects of Treatments by Analyzing the Soaking Water

#### Color, Dry Matter, and Total Color Difference (ΔE*ab) Measurement of the Soaking Water

**Figure 8:**
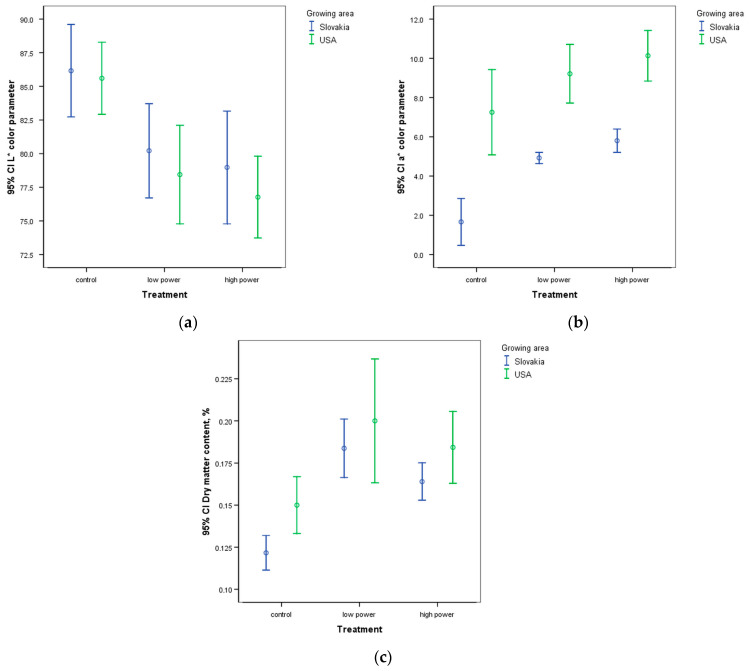
Color parameters (L* (**a**) and a* (**b**)) and dry matter values (**c**) for each treatment after soaking to SRV 2.

Following an investigation into the hydration process, further measurements were conducted to examine the impact of ultrasound treatment (Experiment 2). In this experiment, the beans were soaked only until they reached twice their initial weight (SRV 2), which is the point when they were ready for processing. This was done for the purpose of further studying the effect of ultrasound on the beans. Figure 8 illustrates the results of the color and dry matter measurements. For both origins, the L* parameter of the soaking water decreased due to hydration (distilled water was the initial water), indicating the dissolution of water-soluble components in the soaking water during the process and resulting in a darkening of the water. A significant difference (*p* < 0.05) was observed in the color parameters L* of the soaking water (Figure 8a) between the US-treated groups and the control groups for samples from both growing areas.

However, no measurable difference was found between the groups treated with different ultrasonic parameters. With regard to the a* color parameter (Figure 8b), it is notable that the a* values of samples from the two growing sites displayed a significant difference for all treatments. It was observed that the values of the control USA group were not significantly different from those of the HP-treated Slovakian group. With regard to the a* parameter, no significant difference was observed between the control and the low-power group for the USA group. Only the high-power group differed from the control. In the Slovakian samples, a difference was observed between the treated and untreated groups, but no difference was observed between the two types of ultrasound treatments. With regard to the color parameter b*, no difference was observed between the groups in either the treated or untreated groups or in the origin.

The dry matter content (Figure 8c) of the soaking water was found to show a significant difference between the treated and untreated groups for samples from both growing areas. The dry matter content of the US-treated groups was found to be higher (0.16–0.19%) than that of the conventional soaking groups (0.14–0.15%). No significant difference was observed in the dry matter content between the LP and HP groups.

Table 3 presents the values of ΔE*ab, which indicates whether the difference in the color of the soaking water in the various groups is visible to the human eye. The upper section of Table 3 presents the values in comparison to the control group, while the lower section displays the values in comparison to the low-power group. In accordance with the scientific interpretation of ΔE*ab [40], a value exceeding 2.3 signifies a discernible color difference, even to an untrained observer. It can be observed that the color of the soaking water of the US-treated groups was greater than 2.3, indicating a discernible distinction between the soaking water obtained from conventional and US-assisted soaking. An examination of the values between the two types of US treatment indicates that the Slovakian samples have a measured value just below 2.3, signaling that there is no discernible difference to the human eye. Nevertheless, a distinction can be observed between the LP- and HP-treated groups for the USA samples.

The results of the ΔE*ab parameter indicate that the ultrasonic treatment resulted in a pronounced difference concerning the color of the soaking water in comparison to the color of the soaking water obtained through conventional soaking. This is due to the enhanced release of colorants from the peel caused by the ultrasonic treatment-induced cavitation. However, further studies are required to determine the influence of this phenomenon on the color of the final product.

## 4. Discussion

Food canning represents a significant component of the global food industry, serving a key function in supporting the food supply chain. Among pulses, beans provide an excellent source of vegetable proteins, and they also contain a number of other valuable ingredients, including various minerals. The initial stage of the processing of beans is the hydration of the dry bean seeds. The process is relatively time-consuming. Consequently, research is being conducted to identify potential methods to accelerate it.

Ultrasound represents a potential environmentally friendly and efficacious method for this purpose. The soaking process is subject to a number of influencing factors, including the quality of the soaking water and the temperature. The work had a number of stated aims. The objective was to understand the hydration process of red kidney beans of varying sizes and origins. A model was used to determine the soaking time required for processability, comparing different size groups and treatments.

The results demonstrated that, despite differing surface areas, the soaking times for beans with lengths of 13–15, 15–17, and 17–19 mm were not significantly different from one another for any of the treatment groups (control, low power (LP)—180 W, 20 kHz; high power (HP)—300 W, 40 kHz). This suggests that within this size range, the soaking process can be performed in a uniform technological process, eliminating the necessity for presorting. However, the experiment does not allow for the determination of what size difference causes different soaking times.

The impact of the various treatments was evaluated, and it was determined that the application of ultrasound to both low-power (LP) and high-power (HP) parameters resulted in a notable reduction in the requisite soaking time for processing when compared to the untreated control group. The control group’s soaking time of approximately 4 h can be reduced by approximately 1.5 h, which may represent a significant time saving in the production process. The reason for this effect is that the application of ultrasound improves the mass transfer in the food product [60].

Furthermore, the hydration process characteristics of samples of the same variety from different growing areas were examined. It was found that the time constant describing the hydration dynamics for the samples investigated (origin: Slovakia and USA) and the model-fitted final value (SRV final) differed significantly between the two growing regions. However, the time to processability (SRV2) was not affected by the growing area (except for the HP group). These findings are consistent with those reported in other studies, where the application of ultrasound treatment was demonstrated to enhance the efficiency of cooking operations of chickpeas and rice. In both instances, the moisture absorption rates were observed to increase in accordance with the increase in ultrasonic power [61,62].

NIR spectroscopy represents efficient technology for the analysis of food ingredients. A further objective of our research was to monitor the soaking process using NIRS technology concerning compositional changes. The hydration process was observed with the objective of investigating the characteristics of soaking water obtained during this process. Results indicate that NIRS, combined with chemometric tools, can be used to predict soaking water characteristics with high accuracy. PLSR- and SVR-based modeling on dry matter content and lightness color parameters were highly accurate and robust, potentially capturing chemical information in samples.

Regression and loadings vector analysis revealed the presumed presence and contribution of carbohydrates, phenolic, and nitrogenous compounds in regression-based modeling, proposing the chemical composition of the samples. Additionally, it was also suggested that samples from beans of varying sizes and origins could be combined to develop more comprehensive and industrially applicable models.

In previous studies, NIRS has been employed in plant breeding studies to examine a range of seed quality attributes in diverse plant species of agronomical interest. These components include protein, oil, fatty acids, acid detergent fiber, and glucosinolates, all of which are affected during the processes of soaking and cooking red kidney beans [63]. The application of ultrasound treatment technology has been demonstrated to induce structural and conformational alterations of food proteins [64] and carbohydrates [65] as a consequence of the physical forces generated during the cavitation process [66].

The nutritional implication of this needs further investigation to assess nutrient losses due to leaching during ultrasound-assisted soaking and the effects of the treatment on the digestibility of major components. To assess the applicability of NIRS, further investigation of the soaking water should be performed by building models specifically on the major chemical compounds extracted. NIRS has been successfully applied to evaluate protein, lipids, tannins, and phytic acid content in bean flour samples in the past [67]. These findings suggest the potential for monitoring the ultrasound-facilitated soaking process by means of rapid assessment of the constituents extracted from beans using NIRS and the appropriate chemometric tools.

The findings of this study indicate that near-infrared (NIR) spectroscopy is a reliable method for predicting the dry matter content of beans during the soaking process. However, the estimation of the color parameters of the soaking water led to only moderate results. It is evident that the industrial application of NIR spectroscopy to monitor the soaking process represents a promising area of research. Nevertheless, in order to verify its applicability, it is essential to conduct a series of additional experiments to quantify the leaching dynamics of individual substances. Furthermore, additional research is necessary to determine the impact of ultrasonic treatment on the structure and color of the final product.

In conclusion, the objective of our research activity was to investigate the compositional changes of the soaking water obtained during the hydration process. It was observed that during the soaking process, the dry matter content of the soaking water increased, and the water became darker and redder in color. Significant differences were observed in these parameters between the US-treated and untreated groups.

The experiments employed two green technologies: US treatment and near-infrared spectroscopy. The application of ultrasound treatment during the soaking process has the potential to significantly reduce the time required for the soaking of dry pulse crops, thereby reducing the time required for industrial production and consequently accelerating the overall production process. The NIRS method offers the advantage of non-invasive analytical measurement, which can be readily incorporated into the digital process, thus rendering it suitable for real-time measurement during and after production. This enhances the quality-control process. This research brings together these emerging technologies to examine their potential for enhancing soaking efficacy and non-invasive, real-time monitoring of the process, which is relevant for both scientific investigative purposes and industrial applications.

## 5. Conclusions

In summary, the ultrasound (US) treatment can significantly accelerate the soaking process. However, the US treatment has been observed to result in the release of greater quantities of water-soluble components from the beans, as evidenced by the darkening of the soaking water’s color, the increase in the a* color parameter, and the rise in the dry matter value. The study does not specify which substances were dissolved by the applied treatment. Further investigation of this area would be beneficial to this research area when considering the applicability of ultrasonic treatment. Nevertheless, the increase in the red color of the soaking water indicates that the ultrasonic cavitation may result in the dissolution of a greater quantity of colorants from the bean peel into the soaking water during the US-assisted soaking than during the conventional soaking process. Further investigation is required to ascertain whether this alteration in color impacts the final product or not.

It has been found that the applied ultrasonic treatments (20 kHz, 180 W, and 40 kHz, 300 W) made it possible to shorten the soaking time of red kidney beans and, therefore, reduce the energy required for the pretreatment process.

NIR spectroscopy analysis was found to be an efficient tool for estimating the components transferred into the soaking water during the ultrasonic treatment-induced soaking process of beans. The approach established here could be applied in the near future to optimize preparatory operations in industrial bean processing, thereby reducing processing costs.

## Figures and Tables

**Table 1:** Calculated time to reach SRV2 for beans of varying size, based on the fitted curve for the soaking process.

	Time in min to Reach SRV2		
Treatment	13–15 mm	15–17 mm	>17 mm
USA Origin			
Control	223.82 ± 14.33	257.69 ± 20.31	245.77 ± 18.79
Low power	149.96 ± 4.01	146.44 ± 10.43	148.47 ± 13.27
High Power	146.54 ± 5.79	145.15 ± 11.11	147.76 ± 4.06
Slovakian Origin			
Control	193.01 ± 62.97	204.37 ± 49.90	250.86 ± 31.97
Low power	147.08 ± 3.267	127.99 ± 3.267	132.45 ± 24.74
High Power	113.26 ± 18.77	131.59 ± 15.32	114.77 ± 4.32

**Table 2:** Model metric summary for the prediction of soaking water parameters. Savitzky–Golay filter with second-order polynomial and 21 smoothing points as pre-treatment for all models. Values are averages with standard deviations based on the three different test-set samplings.

	Dry Matter Content		L*		a*		b*	
Method	PLSR	SVR	PLSR	SVR	PLSR	SVR	PLSR	SVR
Obs. train	293 ± 0	293 ± 0	293 ± 0	293 ± 0	293 ± 0	293 ± 0	293 ± 0	293 ± 0
Obs. pred.	73 ± 0	73 ± 0	73 ± 0	73 ± 0	73 ± 0	73 ± 0	73 ± 0	73 ± 0
Comp.	5.333 ± 0.471	12.667 ± 0.471	5.333 ± 0.471	12.667 ± 0.471	5 ± 0	3.667 ± 0.471	5 ± 0	18.333 ± 3.771
RMSEC	0.01 ± 0.001	0.01 ± 0	2.605 ± 0.083	2.53 ± 0.024	2.289 ± 0.037	2.117 ± 0.338	4.473 ± 0.034	4.486 ± 0.044
R^2^C	0.947 ± 0.012	0.95 ± 0.003	0.866 ± 0.009	0.873 ± 0.002	0.58 ± 0.015	0.629 ± 0.122	0.588 ± 0.007	0.585 ± 0.008
RMSECV	0.011 ± 0.001	0.011 ± 0	2.89 ± 0.061	2.798 ± 0.013	2.474 ± 0.047	2.544 ± 0.185	4.926 ± 0.037	5.027 ± 0.047
R^2^CV	0.934 ± 0.013	0.935 ± 0.004	0.835 ± 0.007	0.845 ± 0.001	0.506 ± 0.018	0.475 ± 0.077	0.5 ± 0.008	0.479 ± 0.01
RMSEP	0.011 ± 0.001	0.01 ± 0	2.559 ± 0.136	2.546 ± 0.122	2.395 ± 0.134	2.221 ± 0.349	4.507 ± 0.071	4.546 ± 0.167
R^2^P	0.933 ± 0.012	0.951 ± 0.008	0.869 ± 0.013	0.87 ± 0.012	0.532 ± 0.049	0.588 ± 0.134	0.573 ± 0.015	0.564 ± 0.032

**Table 3:** Examination of the effect of various treatments on color change using the ΔE*ab value. The results presented in the upper part of the table were calculated in comparison to the color of the control group, while the results in the lower part were calculated in comparison to the LP group.

**Treatment**	**Growing Area**	ΔE*ab Compared to the Control **(mean ± SD)**
Low power	Slovakian	7.18 ± 1.54
	USA	8.79 ± 3.46
High power	Slovakian	7.41 ± 0.74
	USA	11.85 ± 3.85
	**Growing Area**	ΔE*ab Compared to the Low Power (LP) Value (mean ± SD)
	Slovakian	2.27 ± 1.86
	USA	5.22 ± 3.01

## Data Availability

Data are contained within the article.
